# Effect of fascia iliaca compartment block combined with ropivacaine on post-operative outcomes in elderly patients undergoing hip fracture repair

**DOI:** 10.12669/pjms.40.4.8283

**Published:** 2024

**Authors:** Junshi Li, Hui Chen, Jianfeng Zuo, Xiping Zhang

**Affiliations:** 1 Junshi Li, Department of Anesthesiology, Changxing County People’s Hospital, Changxing Country, Huzhou 313100, Zhejiang Province, P.R. China; 2 Hui Chen, Department of Anesthesiology, Changxing County People’s Hospital, Changxing Country, Huzhou 313100, Zhejiang Province, P.R. China; 3 Jianfeng Zuo, Department of Anesthesiology, Changxing County People’s Hospital, Changxing Country, Huzhou 313100, Zhejiang Province, P.R. China; 4 Xiping Zhang, Department of Anesthesiology, Changxing County People’s Hospital, Changxing Country, Huzhou 313100, Zhejiang Province, P.R. China

**Keywords:** Fascia iliaca compartment block, Hip fracture, Ropivacaine

## Abstract

**Objective:**

To explore the effect of fascia iliaca compartment block (FICB) in combination with ropivacaine on post-operative outcomes in elderly patients undergoing hip fracture (HF) repair.

**Methods:**

Retrospective analysis included data of 111 elderly patients who underwent HF surgery with FICB in Changxing County People’s Hospital from October 2018 to October 2022. Observation group received 0.25% ropivacaine combined with FICB (n=52), and the control group was administered an intravenous injection of parecoxib sodium (n=59). Baseline characteristics of the patients, and indexes such as mean arterial pressure (MAP), heart rate (HR), and visual analogue scale (VAS) pain scores, were collected at one-, six-, 12- and 24-hours past surgery, both at rest and after passive movement.

**Results:**

VAS scores, MAP and HR at rest and after a passive movement in both groups were comparable before the surgery. VAS sores were significantly lower in the observation group at one-, six-, 12- and 24-hours after the surgery (P<0.05). Postoperative MAP in the observation group (80.83 ± 8.31) was significantly lower compared to the control group (95.29 ± 8.45 (t = -9.0659, p < 0.0001). Similarly, HR of the observation group was significantly lower one-hour post-surgery both at rest (t = -2.0468, p = 0.0431) and after passive movement (t = -6.0625, p < 0.001), and at all subsequent time intervals after the passive movement (P<0.05).

**Conclusions:**

Ropivacaine combined with FICB was associated with improved post-operative outcomes such as lower post-surgery VAS scores, MAP and HR compared to the intravenous injection of parecoxib sodium.

## INTRODUCTION

Hip fractures (HFs) are common orthopedic events in clinical settings, especially in elderly population.^1^ About 95 % of all HFs are due to falls^2^ with the estimated annual global prevalence of 4.5 million by 2050.^3^ HFs are particularly dangerous in the geriatric patients, with high (nearly 30%) mortality within a year after the fracture. Moreover, due to the frailty, elderly HF patients are more seriously affected by pain, mobility problems and inability to take care of themselves.^4^

While conservative treatment such as oral analgesia, bone traction, bracketing, etc^2^ can relieve local pain and help patients recover some of their mobility, they have an overall poor effect. Loss of mobility in geriatric HF patients is associated with complications such as pneumonia,^5^ pressure ulcers,^6^ deep venous thrombosis,^7^ etc. Therefore, in the developed countries with a high income, most HFs are treated surgically, with hospitalization and subsequent rehabilitation.^1^ Spinal anesthesia is routinely used for HF surgery since it is associated with reduced operative time, less bleeding, and lower rate of complications.^8^,^9^ However, the choice of anesthesia for elderly hip fracture surgery remains controversial.^10^

Elderly patients with HF struggle to maintain the position required for proper spinal anesthesia. Recently, the use of fascia iliaca compartment block (FICB) before spinal anesthesia became increasingly popular method of fast pain relief not only for reducing the positioning pain but also for lowering the required puncture time of spinal anesthesia.^11^,^12^ FICB involves a single injection of local anesthetic immediately dorsal to the fascia iliaca,^13^,^14^ which effectively blocks the obturator, femoral, and lateral femoral cutaneous nerves in the iliac fossa.^4^ Since elderly patients with diminished physical functions often have poor tolerance to anesthetics, it is crucial to select appropriate anesthesia choices for this population.^15^,^16^ Bupivacaine, the most widely used long-acting regional amide anesthetic, has been associated with certain cardiotoxicity.^17^ Ropivacaine is a stereo-specific levorotary local anesthetic that acts as a vasoconstrictor. It has a lower cardiac toxicity risk, and longer-lasting effect,^18^ making it an effective anesthetic for FICB.^19^ In patients undergoing lower limb surgery, ropivacaine was comparable to bupivacaine in terms of its anesthetic profile.^17^ Currently, the selection and dosage of local anesthetic agents for elderly HF patients are determined by anesthesiologists. Since these medications directly affect the anesthesia effect, hemodynamics of the patients, and the outcomes, it is crucial to evaluate the effect of available local anesthetic options on postoperative outcomes in this population of patients.

This study aimed to evaluate the effect of ropivacaine in combination with FICB on post-operative outcomes in elderly patients. Our results may be used in clinical decision-making to determine the safest and most efficient regional anesthesia scheme for geriatric patients undergoing hip fracture repair.

## METHODS

Clinical records of 111 elderly patients (46 males and 65 females) who had undergone FICB for HF surgical repair in Changxing County People’s Hospital from October 2018 to October 2022, were retrospectively analyzed. Of them, 52 patients received FCIB in combination with ropivacaine (observation group), and 59 patients received intravenous injection of parecoxib sodium (control group), a parenteral COX-2 inhibitor that is routinely used for reducing postoperative pain.^20^ The average age of patients was 71.01±5.30 years. The mean time from injury to operation was 3.07±1.45 days. Of 52 patients in the observation group, 36 were classified as presenting American Society of Anesthesiologists (ASA) II grade and 16 as having ASA III grades. Of 59 patients in the control group, 36 were ASA II grade and 23- ASA III grade.

### Inclusion criteria:

- Age over 60 years old
- ASA score of II-III
- X-ray confirmation of unilateral HF.^21^
- Patient with surgery and anesthesia indications.
- Patient with complete clinical data.

### Exclusion criteria:

- Patients with peripheral neuropathy.
- Patients with multiple fractures.
- Patients with a history of chronic pain.
- Patients on long-term psychotropic drugs.
- Patients with infection at the puncture site of the fascia iliaca compartment.

### Ethical Approval

The Medical Ethics Committee of Changxing County People’s Hospital approved this study (approval number, 2023-04-11; date, 2023-04-19).

Patients in the observation group received 0.25% ropivacaine combined with FICB. The treatment method was as follows, and all procedures were performed by one experienced anesthesiologist. The FICB was done under the guidance of ultrasound. The patient was placed in a supine position. The anesthesiologist disinfected the skin, and placed a 6-13 MHz high-frequency probe (Sonosite HFL38, USA) along the inguinal fold and inserted the needle. The puncture point was located at the junction of the middle and outer 1/3 of the line between the anterior superior iliac spine and the pubic tubercle, 2 cm caudally. Patients in the observation group were injected with 2mL of 0.25% ropivacaine hydrochloride (Hebei Yipin Pharmaceutical Co., Ltd; Approval No. 20113463), and patients in the control group received injection of 40 mg parecoxib sodium after verifying the absence of blood return. With the procedure done properly, the ultrasound guided beam should show caudal-to-cephalad structures in the plain between the internal oblique and iliacus muscles. Then, 30ml of 0.25% ropivacaine was injected in a way that pushed the iliac fascia away from the inside out from the injection point. After the drug spread into the iliopsoas muscle or above the iliac fascia, the needle position was adjusted. The block was considered effective if the patient complained of significant pain relief after injection. After the block was completed, the electronic analgesic pump was connected for continuous administration. The background dose (0.125% ropivacaine) was set at 7mL per hour. The patient-controlled analgesia was set at 5 mL and the locking time at 15 minutes.

Electrocardiogram (ECG), blood oxygen saturation, blood pressure, and heart rate values were monitored in the operating room. Values for the mean blood arterial (MAP) and heart rate (HR) were measured and recorded before anesthesia induction, at the end of the operation, and 24 hours after. The analgesic and sedative effects were evaluated before analgesia (T0), 12 hours after the operation (T1), 24 hours after it (T2), and 48 hours after (T3). Visual analog pain (VAS) scores were used to evaluate the pain relief (10 points for severe pain and zero points for absence of pain).^22^

### Statistical analysis

Data analysis was performed using STATA version 17. For categorical variables, frequency distributions were provided and presented as percentages. For continuous variables, mean and standard deviations (SD) were calculated. Independent Samples t-test was used for comparing the means of two independent samples, particularly for continuous variables. The assumption of equal variances was checked and considered in the analysis. The Chi-square test was used to compare categorical variables like gender distribution and the presence of diabetes between the two groups. A p-value less than 0.05 was considered indicative of a statistically significant association or difference.

Repeated measures analysis was carried out to assess the VAS scores, MAP, and HR at various time intervals post-surgery, considering both ‘at rest’ and ‘after passive movement’ states. A repeated measures ANOVA (analysis of variance) was used to determine within-group differences over time, while an independent samples t-test was applied for between-group comparisons at each time interval. Before performing the t-tests, the normality of data distribution was checked. In cases where the data were not normally distributed, appropriate non-parametric tests would have been considered. However, given the provided results, it is assumed that the data met the required assumptions for the t-test. The results were considered statistically significant at a p-value threshold of <0.05.

## RESULTS

This retrospective study included clinical records of 111 patients (46 males and 65 females). Of them, 52 patients received ropivacaine combined with iliac fascia space block (observation group), and 59 patients were administered an intravenous injection of parecoxib sodium needle for analgesia (control group). As summarized in Table-I, there was no significant difference in gender distribution, incidence of diabetes, ASA classification, body mass index, drinking history, and age between the groups (P>0.05).

**Table-I:** Comparison of demographic characteristics between Observation (n=52) and Control group (n=59).

Variables/Characteristics	Observation group (n=52)	Control group (n=59)	t/χ2 value	p-value
***Gender (Male/Female)***				
Male	24 (46.15%)	22 (37.29%)	0.8952	0.344
Female	28 (53.85%)	37 (62.71%)		
Diabetes (Yes/No)				
No	32 (61.54%)	41 (69.49%)	0.7765	0.378
Yes	20 (38.46%)	18 (30.51%)		
ASA classification				
II level	36 (69.23%)	36 (61.02%)	0.8182	0.366
III level	16 (30.77%)	23 (38.98%)		
Drinking history (Yes/No)				
No	38 (73.08%)	39 (66.10%)	0.6329	0.426
Yes	14 (26.92%)	20 (33.90%)		
Age (in years) Mean±SD	71.79±5.26	70.85±5.54	0.9142	0.3627
BMI (kg/m^2^) Mean±SD	23.20±2.67	23.89±2.53	-1.4081	0.1619

***Notes:*** The values in parentheses for categorical data are percentages. The values after “±” for continuous data are standard deviations.

### VAS Scores

Postoperative VAS scores at rest and after passive movement were significantly different between the two groups (Table II). While the VAS score at rest before the surgery was similar in both groups (t = 1.4528, p = 0.1492), the observation group demonstrated a significantly lower VAS score at rest 1h (t = -2.5544, p = 0.0120), 6h (t = -2.1861, p = 0.0309), 12h (t = -2.4089, p = 0.0177), and 24h post-surgery (t = -2.3787, p = 0.0191). A similar trend of significant lower scores for the observation group was seen after passive movement 1, 6, 12 and 24h after the surgery (Table-II).

**Table-II:** Comparison of VAS score at rest and after passive movement between Observation (n=52) and Control group (n=59).

VAS score at different time points	Observation Group	Control Group	t/F value	p value
VAS at rest before surgery	5.88±1.13	5.57±1.10	1.4528	0.1492
VAS at rest one-hour post-surgery	3.90±1.14	4.44±1.07	-2.5544	0.0120
VAS at rest six hours post-surgery	3.09±1.17	3.61±1.29	-2.1861	0.0309
VAS at rest 12 hours post-surgery	2.44±1.24	2.98±1.12	-2.4089	0.0177
VAS at rest 24 hours post-surgery	2.75±1.10	3.24±1.06	-2.3787	0.0191
Within group difference for VAS at rest			325.72	<0.001
VAS at passive movement before surgery	7.98±1.15	8.07±1.14	-0.3999	0.6900
VAS at passive movement one-hour post-surgery	4.35±1.17	6.24±1.41	-7.6430	<0.001
VAS at passive movement six hours post-surgery	4.11±0.92	5.83±1.19	-8.3996	<0.001
VAS at passive movement 12 hours post-surgery	3.96±0.07	5.61±1.16	-7.9379	<0.001
VAS at passive movement 24 hours post-surgery	4.21±1.13	5.91±1.29	-7.3642	<0.001
Within group difference for VAS at passive movement			440.85	<0.001

### MAP Measurements

There were no differences in the mean arterial pressure (MAP) at rest and after passive movement between both groups before the surgery. One-hour post-surgery, MAP after passive movement, was significantly lower (80.83 ± 8.31) in the observation group compared to the control group (95.29 ± 8.45; t = -9.0659, p < 0.0001). Similarly, at all subsequent time intervals after passive movement, MAP was significantly lower in the observation group compared to the control group (Table-III).

**Table-III:** Comparison of MAP at rest and after passive movement between Observation (n=52) and Control group(n=59)

Variable	Observation Group (Mean ± SD)	Control Group (Mean ± SD)	t value/ F-value	p value
MAP at rest - Before analgesic operation	78.25 ± 6.69	79.15 ± 5.84	-0.7592	0.4494
MAP at rest - One hour post surgery	79.44 ± 6.79	80.34 ± 6.42	-0.7150	0.4761
MAP at rest - Six hours post surgery	80.46 ± 6.11	82.05 ± 7.86	-1.1780	0.2414
MAP at rest - 12 hours post surgery	79.87 ± 6.53	81.71 ± 7.33	-1.3940	0.1662
MAP at rest - 24 hours post surgery	80.17 ± 7.68	82.22 ± 7.99	-1.3721	0.1729
Within group difference for MAP at rest			8.03	<0.001
MAP after passive movement - Before analgesic	87.38 ± 6.88	87.80 ± 7.59	-0.2979	0.7663
MAP after passive movement - One-hour post-surgery	80.83 ± 8.31	95.29 ± 8.45	-9.0659	<0.0001
MAP after passive movement - Six hours post-surgery	78.48 ± 8.43	88.47 ± 8.47	-6.2151	<0.0001
MAP after passive movement - 12 hours post-surgery	81.79 ± 9.09	86.71 ± 8.35	-2.9744	0.0036
MAP after passive movement - 24 hours post-surgery	83.96 ± 9.64	88.66 ± 9.07	-2.6449	0.0094
Within group difference for VAS after passive movement			35.19	<0.001

***Note:*** The values mentioned in the Observation Group and Control Group columns are in the format Mean ± Standard Deviation (SD).

### Heart Rate Comparisons

A comparison of preoperative heart rate (HR) at rest and after passive movement did not detect significant differences between the groups (Table-IV). Patients in the observation group had statistically significant lower HR one-hour post-surgery both at rest (t = -2.0468, p = 0.0431) and after passive movement (t = -6.0625, p < 0.001). This pattern was consistent for all subsequent time intervals after passive movement (Table-IV).

**Table-IV:** Comparison of heart rate (HR) at rest and after passive movement between Observation (n=52) and Control group (n=59).

Variables (Time Interval)	Observation Group (Mean ± SD)	Control Group (Mean ± SD)	t-value/ F-value	p-value
HR at rest - Before analgesic	77.38 ± 9.93	78.32 ± 9.83	-0.4992	0.6187
HR at rest - One hour post surgery	75.87 ± 7.83	78.83 ± 7.42	-2.0468	0.0431
HR at rest - Six hours post surgery	76.19 ± 8.16	79.25 ± 7.80	-2.0190	0.0459
HR at rest - 12 hours post surgery	78.50 ± 7.89	80.63 ± 7.58	-1.4474	0.1507
HR at rest - 24 hours post surgery	80.19 ± 7.56	82.27 ± 7.78	-1.4232	0.1575
Within group difference for HR at rest			27.35	<0.001
HR after passive movement - Before analgesic	98.92 ± 10.14	99.46 ± 11.15	-0.2630	0.7931
HR after passive movement - One hour post surgery	78.31 ± 7.45	86.75 ± 7.20	-6.0625	<0.001
HR after passive movement - Six hours post surgery	79.31 ± 7.27	87.93 ± 10.16	-5.0802	<0.001
HR after passive movement - 12 hours post surgery	78.42 ± 7.76	88.71 ± 9.80	-6.0774	<0.001
HR after passive movement - 24 hours post surgery	82.54 ± 8.19	93.59 ± 9.67	-6.4517	<0.001
Within group difference for HR after passive movement			137.57	<0.001

## DISCUSSION

The results of our study showed that in elderly patients ropivacaine in combination with FICB results in better postoperative outcomes in terms of VAS scores, MAP measurements, and HR especially after passive movement compared to the intravenous injection of parecoxib sodium.

Ultrasound guided FICB can help the anesthesiologist identify the precise drug diffusion site, ensure the success rate of blocks, and facilitate the continuous infusion of analgesic drugs, while improving the oxygen supply between tissues and the blood perfusion around the surgical area.^23^ Ropivacaine is a local anesthetic with a propyl group on the third piperidine nitrogen atom; it blocks sodium ions from flowing into nerve fiber cell membranes, reversibly blocking impulse conduction, hyperpolarizing nerve cells, blocking signal transmission, and producing analgesic and sedative effects.^24^,^25^

Our study demonstrated that while FICB procedure led to transient hemodynamic effect, as indicated by the rise in MAP and HR of the patients, ropivacaine in combination with FICB led to a significant reduction in the MAP and HR of patients after passive movement compared to parecoxib injection (p<0.005). Lower MAP and HR after passive movement were consistent over the extended period and remained significantly lower than that of the control group 24h after the surgery. Our results are consistent with the observation by Taksande K et al. that epidural ropivacaine stabilizes the hemodynamics and reduces the general anesthesia requirements.^26^

Patients who received FICB with ropivacaine had significantly lower VAS scores at rest and after passive movement compared to the control group. Pain scores remained lower at different time points (1, 6, 12 and 24h) after the surgery, indicating that FICBs with ropivacaine produced more efficient analgesic effect compared to the intravenous injection of parecoxib sodium. This is consistent with previous studies. Rosetti J et al showed that ropivacaine had significant analgesic and sedative effects.^27^ A 1998 study by Fanelli et al^28^ demonstrated that orthopedic surgery patients who received combined femoral and sciatic nerve block with ropivacaine had better postoperative pain relief and a longer duration of analgesia than recipients of other analgesics. Regaining mobility after surgery is a top priority in the treatment of hip fractures, especially in elderly patients. Bertini et al.^29^ showed that in patients who had undergone hip arthroplasty ropivacaine resulted in significantly lower incidence of motor block with similarly effective pain relief and overall greater patient satisfaction. Considering these results together with our observation that ropivacaine leads to better analgesic effect, we may speculate that FICB in combination with ropivacaine can allow HF patients to regain their agility sooner after the surgery, thus preventing possible side-effects of immobility. Thus, ropivacaine in combination with FICB can be considered as an effective anesthetic scheme for treating geriatric HF patients.

### Limitations

This was a single center retrospective study with a small sample. This could potentially introduce an unavoidable selection bias. Additionally, we focused on few indicators, which may lead to a certain bias in our results. Observation times in our study were limited to only 24h after the procedure. Further prospective studies with larger sample sizes and longer follow-ups are needed. These studies should evaluate additional indicators that may provide more comprehensive data on the effect of different local anesthetic drugs in elderly patients undergoing FICB.

## CONCLUSION

FICBs with ropivacaine had a beneficial effect for the elderly patients with HF in our cohort, and was associated with improved post-operative outcomes such as lower post-surgery VAS scores, MAP and HR compared to the intravenous injection of parecoxib sodium.

### Authors’ contributions:

**JL:** Conceived and designed the study.

**HC**, **JZ** and **XZ:** Collected the data and performed the analysis.

**JL:** Was involved in the writing of the manuscript and is responsible for the integrity of the study.

All authors have read and approved the final manuscript
